# The Case of the Children's Hospitals

**Published:** 1919-02-22

**Authors:** Isabel Macdonald

**Affiliations:** Secretary, Royal British Nurses' Association.


### THE CASE OF THE CHILDREN'S HOSPITALS.

*To the Editor of* The Hospital.

Sir,—In your issue of February 15 Sir Cooper Perry replies to the letter of the Chairman and the Vice-Chairman of the Hospital for Sick Children, and his remarks in connection with my attitude towards the position of nurses trained in children's hospitals are grossly misleading. I claim the right to explain my position and to criticise his statements.

In the first place, Sir Cooper Perry takes exception to the description by your correspondents of the College Bill as the "draft Bill for the College of Nursing"; but the College Bill may quite fairly be so described; its first provision being that "The College of Nursing, Limited, shall be entitled to bear the title of 'The College of Nursing,' without the addition of the word 'Limited,' and is hereinafter described as the College of Nursing." Further, Clause 5, Section 3, in the Bill reads as follows:—

"The Memorandum and Articles of Association of the College of Nursing shall, so far as they are consistent with this Act, remain in full force and effect unless and until they have been varied or repealed by Rules made under this Act."

Sir Cooper Perry refers to the "rival Bill of the Central Committee," thereby giving the impression that the College of Nursing had a Bill in the field, and that the Central Committee had drafted one in opposition to it. The facts are that previous to 1909 three different Societies had drafted Bills for the State Registration of Trained Nurses. In that year, however, as a result of negotiations between the organised Societies of Nurses, the Central Committee was formed, which, taking as a basis for discussion the Bill which passed the House of Lords in 1908, succeeded, after a certain amount of "give and take" on the part of the various societies, in drafting an agreed Bill, which obtained a large majority when introduced into the House of Commons in 1914 under the ten-minutes' rule. It remained for the College of Nursing, by cutting across its bows with a rival Bill, and one opposed to principles which the nurses' organised societies are pledged to support, to destroy the unity in regard to legislation which the Central Committee had secured by the expenditure of no small amount of money and effort.

Sir Cooper Perry states that the Register of the College, with the exception of "something like ten laymen and a few doctors, is by the Memorandum and Articles of Association of the College of Nursing, Limited, confined absolutely to women trained in general nursing." I find no such restriction in the aforementioned Memorandum and Articles of Association.

Sir Cooper Perry evidently thinks that the College Bill will be more acceptable to the authorities of children's hospitals if it leaves a "loophole" whereby their nurses can have their names placed on supplementary registers—a disingenuous proposal scarcely calculated to appeal to practical minds. He knows the feeling which exists among nurses with general training against supplementary registers, and obviously he anticipates that the authorities of children's hospitals will take advantage of the "loophole" offered to them in the College Bill to press for a Supplementary Register for Children's Nurses as soon as the Bill is placed upon the Statute Book. It is in the lap of the gods whether the General Nursing Council would see fit to "placate" the nurses or the authorities of the children's hospitals. One or other would be doomed to learn a lesson on the folly of putting their trust in permissive legislation.

Sir Cooper Perry states that the children's hospitals find in me "one of the strongest opponents to any Supplementary Register which would admit their nurses to the privileges of registration on any terms." The statement scarcely sounds "British," for Sir Cooper Perry knows that when I opposed the clause in the College Bill taking powers to establish Supplementary Registers, I strongly advocated in place of those a wide system of reciprocity between the general and special hospitals; and, moreover, any one who has read my articles from time to time knows that I am likely to be among the last to minimise the value of training in the nursing of the diseases of children. A scheme of reciprocity such as that which I indicated in my remarks at the meeting would be of the greatest benefit to the nurses, and would add very much to the importance of the special hospitals in any scheme to provide the definite training prescribed by the Council. At the present time a probationer may give three years to training at a special hospital, and at the end of that time find herself disqualified for any of the Government Nursing Services or for the higher posts in the profession, unless she takes another term of three or, it may be, four years' training in a general hospital.

I would point out to Sir Cooper Perry that there was no suggestion at any time to amalgamate the Royal British Nurses' Association with the College of Nursing, Ltd. Under the Supplemental Charter the college was to have been amalgamated with the Association. The Children's Hospitals were not instrumental in defeating the proposed amalgamation. As Sir Cooper Perry well knows, their petition reached the Privy Council subsequent, and not previous, to the insertion of alterations in the Draft Supplemental Charter which, in the opinion of my council, would have seriously affected the ability of the Corporation to keep certain pledges made by the College, and for which it would have become responsible on amalgamating the College with itself.

Sir Cooper Perry concludes with a veiled threat that the Children's Hospitals may find their "loophole" withdrawn from the Bill, adding that its omission will be welcomed by the majority of nurses. Of course it will, but the nurses are not prepared to put much trust in the conscience of a body which, with the one-portal system writ large on one page, proceeds in another to provide for any number of side portals, and which represents to the authorities of the special hospitals that such side portals can be opened at any time, and to the nurses that there is little danger but that they can keep them carefully "banged and barred."—I am, Sir, yours truly,

Isabel Macdonald,
Secretary, Royal British Nurses' Association.

